# Chronic uterine inversion: Mimicking uterine cervical polyp or leiomyoma

**DOI:** 10.1002/ccr3.9288

**Published:** 2024-08-07

**Authors:** Ozer Birge, Hamsatou Saibou, Ilkan Kayar

**Affiliations:** ^1^ Department of Obstetric and Gynecology Maternité de l'Amitié Turqui‐Niger Hospital Niamey Niger; ^2^ Department of Obstetrics and Gynecology Osmaniye State Hospital Osmaniye Turkey

**Keywords:** chronic uterine inversion, leiomyoma, nonpuerperal, uterine cervical polyp, uterine fibroid

## Abstract

**Key Clinical Message:**

The diagnosis of chronic uterine inversion occurs after birth or secondary to pathologies of the pelvic region organs. Especially, the diagnosis and treatment of isolated chronic inversion rapidly under appropriate conditions seem to reduce maternal morbidity and mortality.

**Abstract:**

Chronic uterine inversion is a rare clinical diagnosis and difficult to diagnose and treat. This is a 22‐year‐old patient with no particular history known for a month for uterine fibroid with a polyp, who gave birth through the cervix in the context of subfertility for a year. Non puerperal uterine inversion is a rare clinical condition, and it should be kept in mind that this may be uterine inversion when mass lesions causing clinical complaints are detected, especially in the vulva, vagina, and cervix uteri region. The quality of life of the patients is increased by reducing the morbidity and mortality rates by making a diagnosis with a good clinical and ultrasonographic evaluation.

## 1 INTRODUCTION

Uterine inversion causes serious clinical problems in which the uterus is anatomically inverted, especially the fundus until the cervix is completely inverted out of the uteri. It is a pathological clinical condition that develops in the acute or chronic period, especially during poorly managed labor and/or uterine masses downstream of the cervix and vagina.^1^

Uterine inversion is a rare gynecological pathological condition and has a good prognosis with early diagnosis and appropriate treatment in young women expecting fertility. Nonpuerperal uterine inversion may be related to certain poor living conditions or leiomyoma (56.2% according to Herath et al.^2^).

Chronic uterine inversion is a rare clinical diagnosis and difficult to diagnose and treat. When a literature review was conducted on this subject, it was observed that there were cases that were detected, especially in the puerperal or in the first hours after birth, and it was observed that the case of spontaneously developed chronic uterine inversion without gynecologically isolated polyp or myoma with uterine stem was quite rare.^2^, ^3^ Uterine inversion may be complete or incomplete depending on whether the fundus passes through the cervix.^4^

Chronic inversion can be a secondary consequence of an unrecognized acute inversion or a rare pathology of a submucous myoma, cervical mass, cervical polyp, endometrial polyp, and genial organs prolapsed from the cervix, in any age group. A placental polyp formed by the retention of a cotyledon of the placenta can also cause the same condition. Chronic uterine inversion is a rare clinical diagnosis and difficult to diagnose and treat. Therefore, care should be taken, especially in the case of a vaginal palpable periodic or fragile hemorrhagic mass.

## 2 CASE HISTORY/EXAMINATION

This is a 22‐year‐old patient with no particular history known for a month for uterine fibroid with a polyp, who gave birth through the cervix in the context of subfertility for a year. Her last birth was 3 years ago at home, and it was a normal vaginal birth. Pending (correction of anemia at 7 g/dL of hemoglobin) a surgical program, she came back to us in the context of abundant hydrorrhea and vaginal mass observed after undocumented ablation in an emergency in another center of a polyp being expelled according to the patient.

## 3 METHODS (DIFFERENTIAL DIAGNOSIS, INVESTIGATIONS AND TREATMENT)

**FIGURE 1:**
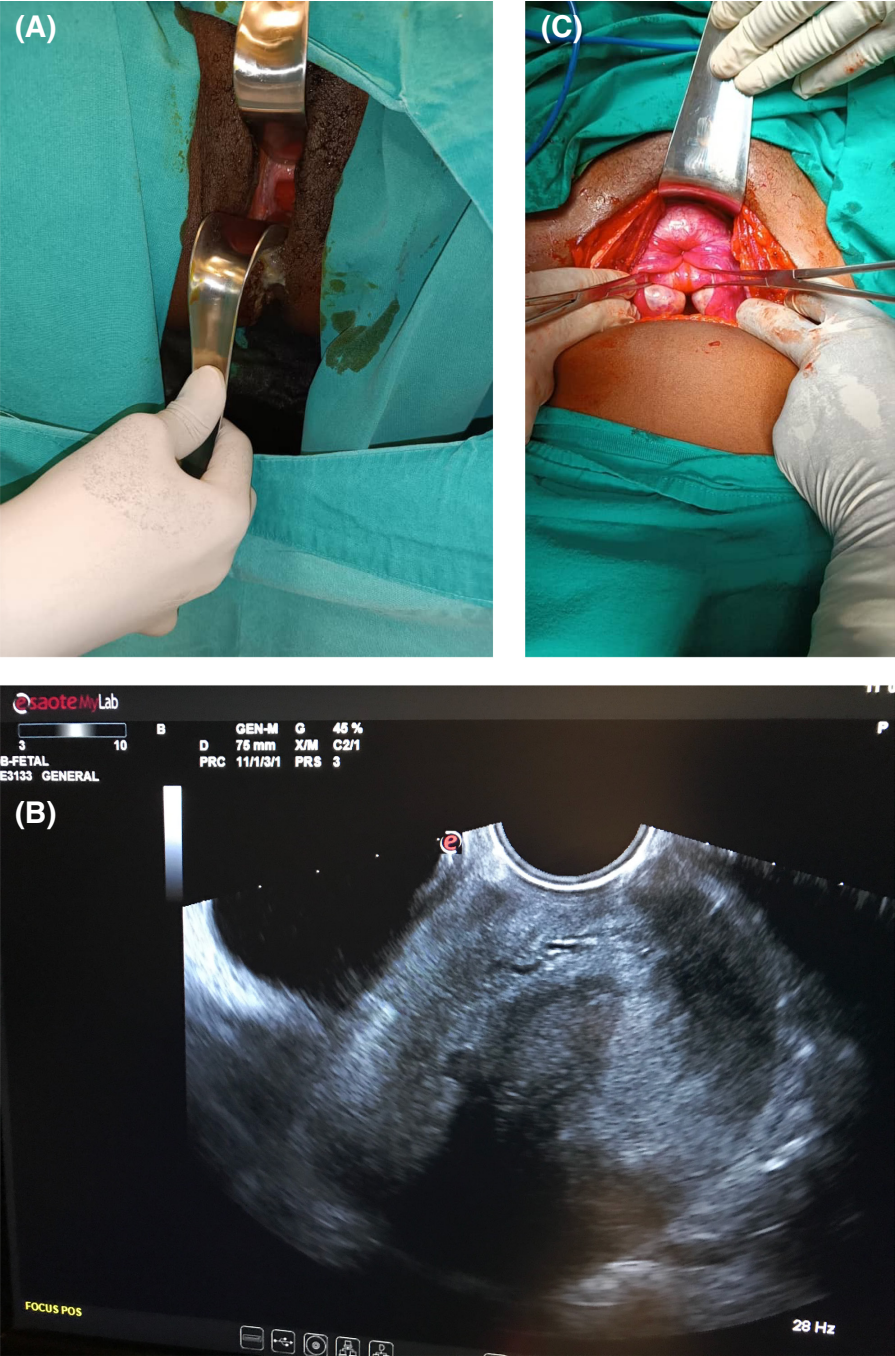
(A) Image of bleeding uterus on preoperative vaginal examination. (B) The uterus is seen to be inverted in the transvaginal ultrasonographic image. (C) Kissing ovaries and tubes in intraoperative pelvic examination (uterus cannot be clearly observed) (flowerwave apperance).

On examination, the absence of a pelvic uterus was noted, and, on the speculum, an oozing reddish cervical mass, palpation of which notes a tight cervical ring behind the mass. A combined abdominal and vaginal attitude followed by repair and repositioning (Houltain's procedure) is preferable among young women (Figure 1A,B, and C). The postoperative follow‐up was simple, with discharge at 3 days and postoperative instructions of sexual abstinence for a month and a half and contraception for a year. At 10 days postoperative, there was a significant decrease in hydrorrhea in the intrapelvic uterus.

Acute uterine inversion is a true obstetric emergency. It can cause serious postpartum hemorrhage. A support team should be called for resuscitation and emergency protocol treatment. In chronic inversion, the uterus involves in an inverted position and is observed in the vagina as a soft, edematous tissue that bleeds easily when touched and shows superficial ulceration areas. In prolonged inversion, the transformation of the uterine wall lined with columnar epithelium into multilayered squamous epithelium can be observed. Chronic inversion is very difficult to correct, partly due to existing edema and the weak structure of the tissues. Techniques used to correct acute inversion are not helpful in this chronic condition. Bed rest, elevating the feet of the bed, antibiotic prophylaxis, and vaginal cleansing with lubricants may help resolve edema and treat any infections. However, eventually a hysterectomy may be required. If the cause of chronic inversion is a myoma or a placental polyp, the polyp should be removed by tying and cutting it at the point closest to the base to facilitate the correction of the inverted uterus.

Further attempts to replace the uterus should be attempted in the operating room under general anesthesia, when conditions and materials are available to perform a laparotomy. Here, first of all, sufficient blood volume and pressure must be provided to correct the patient's vital signs.

## 4 CONCLUSION AND RESULTS

Nonpuerperal uterine inversion is a rare clinical condition, and it should be kept in mind that this may be uterine inversion when mass lesions causing clinical complaints are detected, especially in the vulva, vagina, and cervix uteri region. The quality of life of patients should be increased by reducing disease and mortality rates by diagnosing it with a good clinical and ultrasonographic evaluation in the early period and by starting the appropriate treatment.

## 5 DISCUSSION

Uterine inversion is a condition that can be seen spontaneously (15%–50%) but often develops after birth and requires urgent intervention.^2^, ^3^

Umeononi OS. As a result of the examinations published by Umeononşhu et al.,^5^ a 51‐year‐old multiparous case who had the last normal vaginal delivery 10 years ago and applied to the clinic with inguinal‐abdominal pain, vaginal foul‐smelling discharge, abnormal vaginal bleeding, and a palpable fragile bleeding mass, prolapsed submucosal myoma and advanced stage cervical cancer was detected. After abdominal correction with Haultain's technique, radical hysterectomy and lymph node dissection was performed, and postoperative radiotherapy was planned. Based on this case, it was stated that it should not be forgotten that chronic uterine inversion cases may be accompanied by tumoral lesions in the genital region, especially the cervical and vaginal regions.

A 23‐year‐old young case published by Shivanagappa M. et al.^6^ in November 2013 was brought to the clinic with a poor general condition of shock, and as a result of the history and examination, the patient had a normal delivery 1 year ago and had a palpable mass, abnormal vaginal bleeding, and severe pain that had started a while after delivery. It was learned that she had inguinal pain, and the patient was diagnosed with chronic uterine inversion and complicated sepsis due to uterine pedunculated fibroid, and prompt treatment was arranged, and the importance of careful follow‐up of the cases before and after birth under appropriate conditions was emphasized. In another case report, it was observed that a 35‐year‐old case, who developed serious clinical shock due to excessive vaginal bleeding, developed chronic uterine inversion due to myoma uteri after clinical examination and evaluation, blood product replacement, and intensive care follow‐up. In this case, uterus and fertility preserving surgery was performed with myomectomy and inversion correction using the Houltains method. It has been stated that chronic uterine inversion is a serious, life‐threatening clinical pathology.^7^

Published by Coumary S. et al.^8^ in March 2014 from India, a 65‐year‐old, multiparous postmenopausal case applied to the clinic with a foul‐smelling vaginal discharge that had lasted for 1 month and a bleeding, painful mass protruding from the vagina for 3 months. Although there were no predisposing factors as a result of the examination and tests, a diagnosis of total chronic uterine inversion and total vaginal prolapse was made, a modified Kustner surgical operation was applied to the case, and vaginal hysterectomy and pelvic floor repair were performed vaginally. In conclusion, it was stated that although there was no predisposing factor, it should be kept in mind that inversion and accompanying pelvic organ prolapse can be detected in postmenopausal cases. In another 54‐year‐old postmenopausal case, abdominal hysterectomy was performed in a case of chronic uterine inversion due to a painless fibroma mass hanging down from the vagina for 3 years. In this case, the authors stated that chronic uterine inversion should be kept in mind in case of a palpable mass in the genital area, especially in all age groups from the reproductive age to the postmenopausal age.^9^

Chronic uterine inversion is a much rarer clinical diagnosis and very difficult to diagnose and treat, so care should be taken, especially in cases of a vaginal mass with periodic palpable bleeding or fragile bleeding to touch. When the literature was reviewed on this subject, it was seen that there were cases detected especially in the puerperal or first hours after birth, and it was observed that gynecologically isolated cases of chronic uterine inversion, that is, spontaneously developed without uterine stalked polyps or myomas, were very rare.

Our case gave birth for the first time at an early age under inadequate and unsuitable conditions, and although no secondary pathology was observed in the pelvic region, the development of inversion seems to be due to inadequate postnatal follow‐up and inadequate facilities. The diagnosis of chronic uterine inversion occurs after birth or secondary to pathologies of the pelvic region organs. Especially, the diagnosis and treatment of isolated chronic inversion rapidly under appropriate conditions seem to reduce maternal morbidity and mortality.

## AUTHOR CONTRIBUTIONS

**Ozer Birge:** Resources; software; supervision; validation; visualization; writing – original draft; writing – review and editing. **Hamsatou Saibou:** Resources; validation; writing – original draft; writing – review and editing. **Ilkan Kayar:** Resources; supervision; visualization; writing – review and editing.

## FUNDING INFORMATION

None.

## CONFLICT OF INTEREST STATEMENT

All authors have no conflicts of interests to declare.

## ETHICS STATEMENT

Our institution does not require ethical approval for case reports.

## CONSENT

Written informed consent was obtained from the patient to publish this report in accordance with the journal's patient consent policy.

## Data Availability

The data that supports the findings in this study is available from the corresponding author upon reasonable request.
